# Stress Exposure and the Course of ADHD from Childhood to Young Adulthood: Comorbid Severe Emotion Dysregulation or Mood and Anxiety Problems

**DOI:** 10.3390/jcm8111824

**Published:** 2019-11-01

**Authors:** Catharina A. Hartman, Nanda Rommelse, Cees L. van der Klugt, Rob B.K. Wanders, Marieke E. Timmerman

**Affiliations:** 1 Interdisciplinary Center Psychopathology and Emotion regulation (ICPE), University Medical Center Groningen, University of Groningen, 700 RB Groningen, The Netherlands; c.l.van.der.klugt@student.rug.nl (C.L.v.d.K.); r.b.k.wanders@gmail.com (R.B.K.W.); 2 Department of Psychiatry, Radboud University Medical Center, University of Nijmegen, 6525 GA Nijmegen, The Netherlands; Nanda.Lambregts-Rommelse@radboudumc.nl; 3 Karakter Child and Adolescent Psychiatry, 6525 GC Nijmegen, The Netherlands; 4 Psychometrics & Statistics, University of Groningen, 9712 TS Groningen, The Netherlands; m.e.timmerman@rug.nl

**Keywords:** ADHD, adolescence, stress, emotion dysregulation, anxiety, depression, somatic complaints, multivariate latent growth curve analysis

## Abstract

Background: Compared to typically developing individuals, individuals with attention-deficit-hyperactivity disorder (ADHD) are on average more often exposed to stressful conditions (e.g., school failure, family conflicts, financial problems). We hypothesized that high exposure to stress relates to a more persistent and complex (i.e., multi-problem) form of ADHD, while low-stress exposure relates to remitting ADHD over the course of adolescence. Method: Longitudinal data (ages 11, 13, 16, and 19) came from the Tracking Adolescents’ Individual Life Survey (TRAILS). We selected children diagnosed with ADHD (*n* = 244; 167 males; 77 females) from the TRAILS clinical cohort and children who screened positive (*n* = 365; 250 males; 115 females) and negative (gender-matched: *n* = 1222; 831 males; 391 females) for ADHD from the TRAILS general population sample cohort (total *n* = 1587). Multivariate latent class growth analysis was applied to parent- and self-ratings of stress exposure, core ADHD problems (attention problems, hyperactivity/impulsivity), effortful control, emotion dysregulation (irritability, extreme reactivity, frustration), and internalizing problems (depression, anxiety, somatic complaints). Results: Seven distinct developmental courses in stress exposure and psychopathology were discerned, of which four related to ADHD. Two persistent ADHD courses of severely affected adolescents were associated with very high curvilinear stress exposure peaking in mid-adolescence: (1) Severe combined type with ongoing, severe emotional dysregulation, and (2) combined type with a high and increasing internalization of problems and elevated irritability; two partly remitting ADHD courses had low and declining stress exposure: (3) inattentive type, and (4) moderate combined type, both mostly without comorbid problems. Conclusions: High-stress exposure between childhood and young adulthood is strongly intertwined with a persistent course of ADHD and with comorbid problems taking the form of either severe and persistent emotion dysregulation (irritability, extreme reactivity, frustration) or elevated and increasing irritability, anxiety, and depression. Conversely, low and declining stress exposure is associated with remitting ADHD and decreasing internalizing and externalizing problems. Stress exposure is likely to be a facilitating and sustaining factor in these two persistent trajectories of ADHD with comorbid problems into young adulthood. Our findings suggest that a bidirectional, continuing, cycle of stressors leads to enhanced symptoms, in turn leading to more stressors, and so forth. Consideration of stressful conditions should, therefore, be an inherent part of the diagnosis and treatment of ADHD, to potentiate prevention and interruption of adverse trajectories.

## 1. Introduction

Over the past years, the focus of research on attention-deficit-hyperactivity disorder (ADHD) has shifted towards its developmental course rather than its childhood characteristics. While the significance of ADHD beyond childhood has become clearer [1,2], insight into the factors that explain individual differences over the course of ADHD is rather limited. One important factor in the variable course of ADHD symptoms may be the exposure to chronic stress. Grant and colleagues [3] reviewed the association between stressors and child- and adolescent psychopathology and showed a bidirectional, continuing, cycle of stressors leading to enhanced symptoms, in turn leading to more stressors, and so forth. Although their paper was not focused on ADHD, previous studies have reported that exposure to stress and ADHD are associated (e.g., family conflict, poor home conditions, death of a close family member or friend [4,5]. These studies suggest the existence of a stress–psychopathology–stress cycle underlying ADHD, in which (families of) individuals with ADHD are not only exposed to “bad luck” stress but also generate stress exposure themselves by creating, for example, peer conflict or financial problems. It may be hypothesized that differences in stress exposure are linked to differences in the course of ADHD, with a reduced stress–ADHD–stress cycle in those who gradually remit and an enhanced cycle in those who persist. Importantly, stress exposure and management of stressful circumstances are in part modifiable, offering the potential for clinical intervention. The literature on stress exposure and (persistence of) ADHD is scant. In the present paper, we study whether individual differences in psychosocial stress exposure between childhood and adulthood are associated with the course of ADHD. Further, we study whether such differences are associated with the core symptoms of ADHD, or with co-occurring problems, including reduced effortful control, emotion dysregulation, and internalizing symptoms.

Traditionally, stress–psychopathology relationships are studied in the context of internalizing problems, in particular depression, anxiety disorders, and medically unexplained somatic complaints [6,7,8] rather than ADHD. Therefore, when studying stress-exposure and the course of ADHD, not only the core symptoms of ADHD, but also the classic stress-related anxiety, depression, and somatic complaints need to be considered as possible comorbid outcomes. It may be hypothesized that individuals with ADHD who are exposed to stress are characterized both by a more persistent form of ADHD and by the onset of comorbid internalizing problems alongside this persistent ADHD trajectory [9]. In addition, the effects of stress exposure in children with ADHD may also differ from the classic stress related internalizing problems. Recent accounts of ADHD symptomatology have proposed that emotional regulation problems (e.g., low frustration tolerance, explosive anger) are an important aspect of ADHD [10,11]. Although not studied as such, it is plausible that children with ADHD who are in stressful circumstances may express emotional regulation problems rather than internalizing problems. For example, laboratory experiments indicate that children with ADHD are more emotionally intense and less proficient in anger management during frustrating, stress-inducing tasks relative to children without ADHD [12,13].

In the present study, we investigate the manifestation of differential developmental trajectories, in which individual differences in stress exposure, core ADHD symptoms, internalizing problems, and emotional regulation problems are clustered. We hypothesize that high exposure to stressful life events is related to a more persistent and complex form of ADHD (i.e., multi-problem, including internalizing and emotional regulation problems), while low-stress exposure relates to remitting ADHD over the course of adolescence. We focus on four waves of longitudinal data collected between age 11–19 from a large number of individuals derived from the TRAILS population and clinical cohorts [14]. This developmental period (early adolescence to young adulthood) is characterized by significant normative developmental changes as well as individual differences over the course of ADHD, stress exposure, internalizing, and emotional regulation problems. We apply multivariate latent class growth analysis on repeated measurements of stress exposure, ADHD, internalizing, and emotional regulation symptoms. This approach makes full use of the repeated simultaneous measurement of all variables and identifies subgroups of individuals with trajectories that shift jointly across multiple domains [15]. This allows identification of subgroups of individuals with ADHD who have distinct stress exposure patterns combined with a different course of ADHD and co-occurring problems.

## 2. Methods

### 2.1. Sample

Participants were a subsample from the population (*n* = 2230) and clinic-referred (*n* = 543) cohorts of TRAILS (Tracking Adolescents’ Individual Lives Survey). TRAILS is a prospective study of Dutch adolescents with bi- and triennial measurements since age 11 [14]. Children in the population sample were recruited through schools in urban and rural areas in the North of the Netherlands. Children in the clinic-referred cohort had been referred to the Groningen University Child and Adolescent Psychiatric Outpatient Clinic at any point in their life (20.8% ≤5 years, 66.1% 6–9 years, 13.1% 10–12 years) for consultation or treatment. 

In the present study, we sampled the children with ADHD from the TRAILS clinic-referred cohort. These participants had a preadolescent lifetime diagnosis of ADHD according to the internet version of the Diagnostic Interview Schedule for Children [16,17]) administered face to face by trained interviewers at measurement wave T1. In addition, we made sure that they still had current ADHD symptoms at age 11 as indicated by a score above the 80th percentile derived from the population cohort based on the parent or teacher report (see instruments section below). This yielded 244 children with ADHD from the clinical cohort (*n* = 244; male–female ratio 2:1). 

The TRAILS population sample was divided into screen positives and screen negatives (i.e., the remaining of the sample) using the 90th percentile of the DSM based ADHD scale based on either parent or teacher report. This yielded 365 children from the general population who were screen positive for ADHD. Their average scores on the parent and teacher-rated ADHD problems of current complaints were comparable to the average scores of children diagnosed with lifetime ADHD from TRAILS CC. Likewise, the gender distribution was highly similar (68.4% and 68.5% males, respectively). 

From the participants from the general population sample who were screen negative (*n* = 1873), all male participants (*n* = 831) were included in the current study and we randomly selected 391 of the screen-negative female participants. This yielded a matched sex distribution of 68% male participants in the screen negative subsample (*n* = 1222). Thus, the total of ADHD (*n* = 244), screen positive (*n* = 365), and screen negative participants (*n* = 1222) added up to 1831 participants that were part of the current study.

We used the data from four measurement waves at ages 11, 13, 16, and 19. See detailed information on the demographic characteristics of our sample in supplementary material). TRAILS was approved by the National Dutch Medical Ethics Committee, in accordance with the ethical standards laid down in the 1964 Declaration of Helsinki. Numbers of approval for the TRAILS population and clinical cohorts are, respectively, P00.0246C and T1: P03.1700C, for T1; P03.105C and P05.1638C, for T2; P03.105C and NL21154.042.07, for T3; and NL22114.042.08 and NL40929.042.12, for T4.

### 2.2. Measurements

Supplementary Figure S1 shows an overview of when the below-described instruments were administered.

#### 2.2.1. ADHD

Attention problems and hyperactivity/impulsivity, respectively, were measured at T1, T2, and T3 with items from the parent-rated DSM based ADHD scale of the Child Behavior Checklist (CBCL) [18]. Attention problems were measured by items that tapped concentration problems, failure to finish things, and being easily distracted. Hyperactivity and impulsivity were measured by items tapping the inability to sit still, impulsiveness, and acting without thinking, being loud, and talking too much.

#### 2.2.2. Internalizing problems

Anxiety, depression, and medically unexplained somatic complaints were measured with the DSM based scales of the Youth Self Report [18] at T1, T2, T3, and T4. The anxiety scale comprises items that tap clinging/being too dependent on adults, phobic fears, school fears, being too anxious, nervousness, worries. The affective problems scale includes sadness, loss of pleasure, feelings of guilt, low self-esteem, thoughts of or attempted suicide, reduced energy, tiredness, eating problems, and sleep problems. The somatic complaints scale includes nausea, pains, stomach or bellyaches, vomiting, eye problems, headaches, skin problems. We used the average scale score (ranging from 0–2) for each scale, facilitating comparison across the different problem domains.

#### 2.2.3. Emotion Dysregulation

Irritability was measured at T1, T2, T3, and T4 and was tapped by a subset of items from the aggression scale of the YSR [18] comprising arguing, screaming, mood changes, temper tantrums, irritability. We used the average scale score (ranging from 0–2).

Extreme reactivity was measured by the “not tuned to the social situation” subscale of the parent-rated Children’s Social Behavior Questionnaire [19] at T1, T2, T3, and T4. Items include: overreacting to everything and everyone, drawing excessive attention to himself/herself, showing sudden mood changes, getting angry quickly, staying angry for a long time, being extremely stubborn, difficulties being corrected when having done something wrong, making a fuss over little things, going on and on about things. We used the average scale score (ranging from 0 to 2).

Frustration was measured by a subscale of the Early Adolescent Temperament Questionnaire—Revised (EATQ-R) [20] rated by parents at T1, T3, and T4. This scale measures the extent of being annoyed by other kids, when being criticized, when not being taken someplace he/she wants to go, when having to stop things he/she likes, when people do not agree with him/her, when he/she makes a mistake in school work. The average scores (range 0–5) were rescaled, such that they had the same 0–2 range as the other scores.

#### 2.2.4. Effortful Control

Effortful control also came from the EATQ-R [20] and was rated at T1, T3, and T4 by parents. This scale includes items like the ability to stick with plans and goals, paying close attention when someone tells how to do something, getting started right away on difficult assignments, finishing homework on time, concentrating on a problem. Reversed scored items include: difficulty finishing things on time, stopping in the middle of doing one thing and going off to do something else, putting off working on a project until it is due, difficulty tuning out background noise and concentrating, forgetting what he or she was about to say when interrupted, doing something fun even when supposed to do homework. The average scores (range 0–5) were rescaled such that they had the same 0–2 range as the other scores.

#### 2.2.5. Stress Exposure

Stress exposure was the sum of chronic difficulties that were present as indicated by the parents in an interview (T1) and by means of a questionnaire (T2, T3, T4). These included chronic illnesses or handicaps in the child or of other immediate family member(s), high work pressure at school, problems at home, neighborhood problems, unemployment, financial difficulties, having fewer friends than the child would like, child being bullied, child having enduring conflicts with family member(s), child having enduring conflicts with a person outside of the family, family member(s) having enduring conflicts.

#### 2.2.6. IQ

The Vocabulary and Block Design subtests from the Revised Wechsler Intelligence Scales for Children (WISC-R) administered at T1 were used to estimate full scale IQ for all children [21] and was recoded based on the tertiles in our data into low (IQ < 90), average (90 ≤ IQ ≤ 105), and high (IQ > 105).

#### 2.2.7. Analysis

To identify the developmental course of stress exposure, ADHD, and related psychopathology, we used multivariate latent class growth analysis (LCGA) [15,22]. In this analysis, the ten outcome variables were jointly modeled to identify groups of individuals (latent classes) with trajectories that shift together across these domains: stress exposure, core ADHD problems (inattention, hyperactivity/impulsivity), reduced effortful control, emotion dysregulation (irritability, extreme reactivity, frustration), and internalizing problems (depression, anxiety, somatic complaints). In this way, a limited number of classes were identified where each class has its own trajectory for all outcome variables. It was assumed that those classes captured the most important aspects of the observed individual trajectories of outcome variables.

The trajectories were modeled using a polynomial function of age, thereby accounting for the varying specific ages of adolescents at each of the four measurement waves. The number of polynomials considered was restricted to two, to avoid overfitting. Visual inspection of individual trajectories of the outcome variables suggested linear patterns of change over time for most outcomes, with the exception of stress exposure. For the outcomes with linear trends, we modeled linear trajectories that characterized the level of the first wave (i.e., intercept referring to the age of 11) and the overall increase or decrease over time (i.e., slope). For stress exposure, we observed in a subset of our sample an inverted U-shape with a peak in mid-adolescence. Trajectories of stress within each class were therefore modeled either as a linear trajectory, a curvilinear trajectory, or an inverted U-shape with an optimum between waves 2 and 3. For the latter, we used a polynomial function of age including an intercept and a quadratic function of age minus 14.4. This ensures that the intercept expresses the level of stress exposure at the age of 14.4 years, corresponding with the age with the average highest value of stress, and the quadratic term the levels of decrease around that age. For each class and for each outcome variable, the two polynomial parameters (i.e., intercept and the linear or quadratic term) were estimated. It follows that the classes were distinguished on the basis of the nature of the trajectories for all outcome variables, as expressed by the intercept and the linear or quadratic term (with the quadratic term only possible for the stress exposure).

We determined the optimal number of classes and the function for stress for each class (i.e., linear or quadratic) based on the statistical model fit and the interpretability of the classes. As a statistical index of model fit, we used the Bayesian Information Criterion (BIC), a widely accepted index for latent class models that penalizes model complexity and increased sample size [23,24]. That is, we fitted LCGA models with 1 to 9 classes, and for each number of classes, the possible combinations of linear and quadratic terms for stress (e.g., for 2 classes: 0, 1, and 2 linear terms, and therefore 2, 1, and 0 quadratic terms, respectively). We selected the model with the lowest BIC that had interpretable classes.

To relate the classes identified by the LCGA to individual demographic and clinical characteristics of the adolescents, we used the three-step approach that takes into account the uncertainty of allocating individuals to the most likely class [25]. In the case of a continuous predictor (e.g., IQ), a categorized version was used. For significantly related predictors, we examined how the predictor was related with the classes. All LCGA analyses were performed with Latent GOLD 5.1 [26].

## 3. Results

### 3.1. Model Selection

The lowest BIC was found for the LGCA model with 9 classes (i.e., the maximal number of classes considered), with 3 classes having a quadratic term and 6 classes having a linear term for stress. We selected the LGCA model with 7 classes—3 classes with quadratic terms and 4 classes with linear terms—for stress, because this model was simpler to interpret than the 9 class model and it had the lowest BIC among the 7 class models. Compared to the selected 7 class model, the 9 class model showed a bit more refinement in the trends of adolescents with relatively healthy outcomes but not in children with ADHD. 

### 3.2. Demographics

The demographic and clinical differences among the four ADHD subgroups, as well as between the ADHD groups and the non-ADHD subgroups, were according to expectation and they strongly supported their validity (see supplementary material for an extensive description). In summary, it was found that proportionally twice as many females were in the ADHD internalizing combined type compared to the inattentive, moderate combined and severe combined types. Low IQ and low and average SES were particularly frequent in the three combined ADHD groups. Among the four ADHD groups, stimulants were most often prescribed for individuals in the severe combined and moderate combined type trajectories and less for individuals in the internalizing combined and inattentive type trajectories. The same pattern was observed for specialized and social care use. 

### 3.3. Overall Developmental Trends

Developments over time were properly captured by linear trends, except for the more severe trajectories of stress exposure, that was better described by a curvilinear trend. Overall, stress exposure showed a decreasing trend over time across subgroups but for those groups with a curvilinear trend they peaked during mid-adolescence. Furthermore, there was an overall decrease in ADHD symptoms over the course of adolescence, stronger for hyperactivity/impulsivity than for attention problems. For internalizing problems, somatic unexplained symptoms decreased over time where depression and anxiety showed an increasing trend, stronger in some subgroups than in others. Interestingly, this was not paralleled by changes in emotion dysregulation problems, which overall showed a decreasing trend and in some subgroups remained at high problematic levels. 

### 3.4. Course Differences in Stress Exposure and Symptoms

#### 3.4.1. Developmental Trajectories

**Figure 1:**
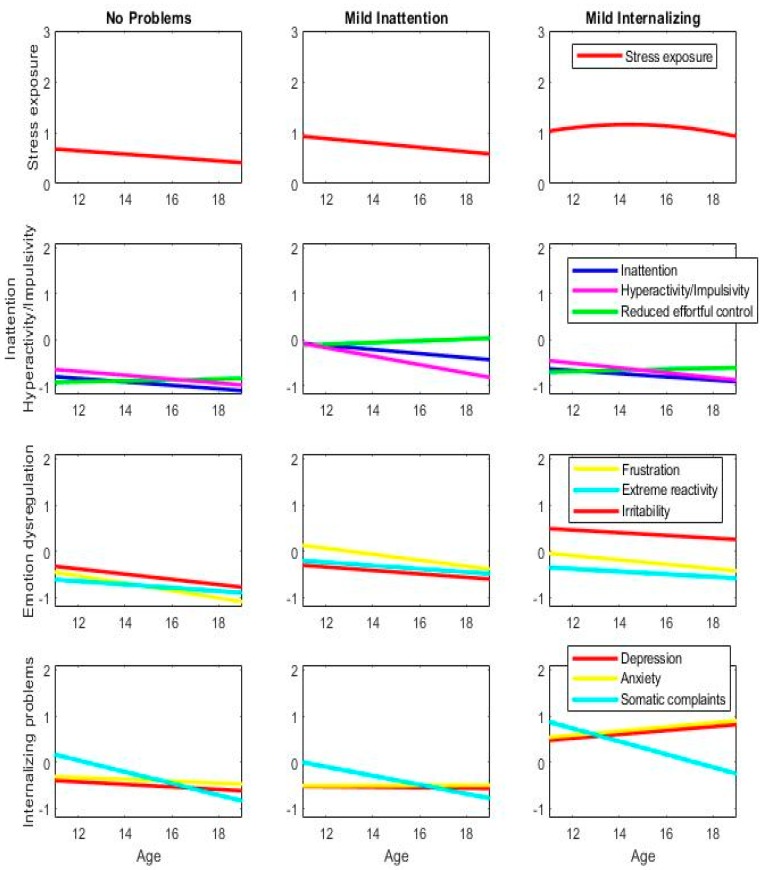

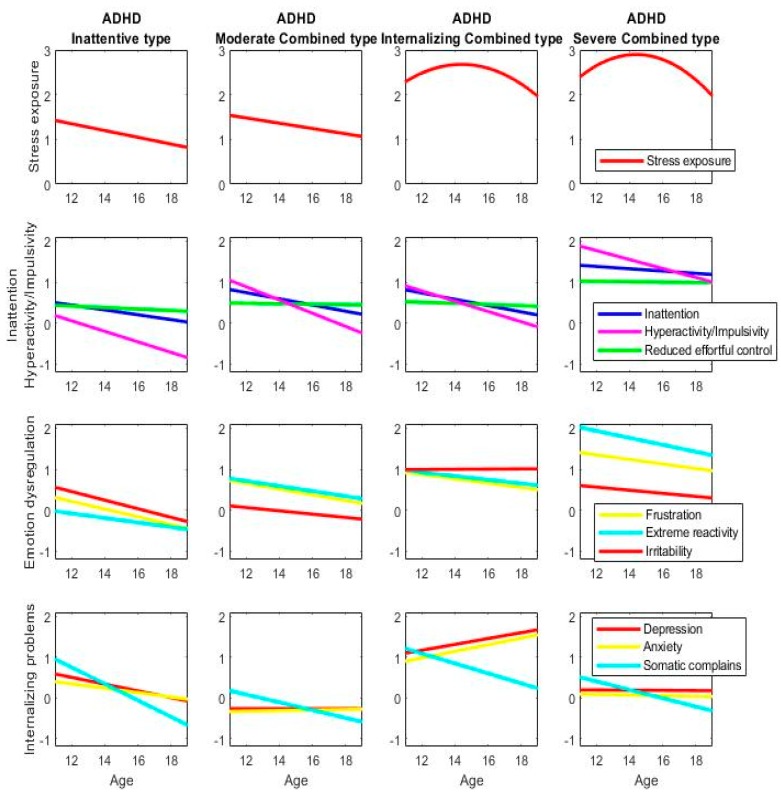
(standardized results). Results of the multivariate latent class growth analysis identifying seven subgroups that differed over the course of stress exposure, core attention-deficit-hyperactivity disorder (ADHD) symptoms, effortful control, and internalizing and emotional regulation problems measured at ages 11, 13, 16, and 19. Unstandardized estimates are plotted for each subgroup (left to right) on trajectories across the four domains (top to bottom).

Seven subgroups were identified that were characterized by differences in the course of stress exposure, core ADHD symptoms, effortful control, and internalizing and emotional regulation problems. The seven subgroups (Figure 1 (standardized scores) and Supplementary Figure S2 (unstandardized scores)) can be divided into three relatively healthy groups and four groups with enhanced levels of ADHD symptoms. Compared to the three non-ADHD subgroups, the four ADHD subgroups had higher levels of stress exposure, parent-rated frustration, extreme reactivity, and reduced effortful control; they were labeled as: “inattentive type,” “moderate combined type”, “internalizing combined type,” “severe combined type.” The three non-ADHD groups showed low levels of ADHD symptoms and were labeled as: “no problems,” “mild inattention,” and “mild internalizing.” Curvilinear stress exposure characterized the latter mild internalizing subgroup and the ADHD subgroups, of the internalizing combined type and severe combined type, with stress peaking in mid-adolescence. The seven subgroups differed significantly in ADHD diagnosis, cohort distribution (i.e., high-risk cohort versus general population cohort), gender, IQ, SES, ethnicity, service use, and psychotropic medication use (all *p* < 0.0001). The associations and characteristics across subgroups are shown in Table 1. 

#### 3.4.2. Stress Exposure and the Course of ADHD

The four ADHD subgroups showed trajectories of ADHD symptoms that differed both in severity and in the course of ADHD over time. 

The inattentive type showed moderate levels of inattention at age 11 with a decreasing trend that remained high together with elevated hyperactivity/impulsivity symptoms at age 11 that reached normative levels in late adolescence. This subtype bears resemblance to the mild internalizing subtype without ADHD symptoms, with comparable parent-reported emotion dysregulation and internalizing problems at age 11 (Figure 1). Despite higher inattention symptoms, the inattentive type had an improving course while the mild internalizing type had a deteriorating course. The inattentive type showed a decreasing trajectory of stress exposure combined with decreasing emotion dysregulation and decreasing internalizing problems, as opposed to a curvilinear stress trajectory that increased in mid-adolescence and increasing internalizing problems in the mild internalizing type. The findings showed that the decreasing, partly normalizing, ADHD symptoms co-occurred reduced stress exposure, which may have protected these individuals against an adverse course of ADHD with further developments of internalizing problems.

The three combined-type ADHD subgroups had increased symptoms of both inattention and hyperactivity/impulsivity and showed poor effortful control. Differences between the subgroups were both in the developmental course of ADHD and co-occurring problems during adolescence. The moderately combined group and the internalizing combined group showed a similar course of core symptoms of ADHD, with a decreasing trend in both attention and hyperactivity/impulsivity symptoms that did not reach normative levels. In contrast, the severe combined type had high persistent ADHD symptoms together with the highest reduced effortful control.

All three combined ADHD types had enhanced emotional regulation problems but differed by the presence and increase of anxiety and depression as well as the extent of ongoing emotion dysregulation problems and trajectories of stress exposure. The emotional regulation problems were lowest for the moderately combined type and differed qualitatively between the internalizing and severe combined ADHD types. When comparing the moderately combined to the internalizing and severe combined ADHD types, the moderately combined type reported lower levels of frustration and irritability and had a decreasing trend of stress exposure together with no depression and anxiety. Both the internalizing combined type and the severe combined type showed strong similarly enhanced trajectories of stress exposure with a curvilinear association over time, peaking in mid-adolescence. However, the internalizing combined type had high levels of stable irritability and the internalizing problems for these individuals were already high at age 12, especially when compared to the other three ADHD types. Similar to the developmental patterns in the mild internalizing type without ADHD symptoms, these were followed by an adverse course of depression and anxiety at later ages. In contrast, the severe combined type showed no adverse trajectories of internalizing problems despite high ADHD symptoms, strongly enhanced stress exposure, and severe emotional regulation problems of irritability, extreme reactivity, and frustration. The combination of high impulsivity, reduced effortful control, and high frustration together with lower irritability and the absence of depression and anxiety characterized a more externalizing profile in this ADHD type. 

## 4. Discussion

Our study examined the hypothesis that high-stress exposure is associated with a poor ADHD course from pre-adolescence to young adulthood. We first showed that stress exposure was consistently higher in the four ADHD subgroups as compared to the non-ADHD subgroups, in line with existing literature [4,5]. Second, the two non-remitting ADHD subgroups were associated with very high, curvilinear stress exposure (peak in mid-adolescence), while the two remitting ADHD subgroups were related to lower as well as declining stress exposure, therefore, supporting our hypothesis. Third, the findings indicated that the two persisting ADHD combined types with very high-stress exposure were associated with either co-occurring severe and persistent emotion dysregulation (irritability, and especially extreme reactivity and frustration) or elevated irritability and elevated and increasing anxiety and depression symptoms. In terms of demographic characteristics, comparatively low SES and low IQ characterized both trajectories, while stimulant use was more likely in the former group, and females were more often present in the latter group. Findings suggest that the stress–psychopathology–stress cycle may play an important facilitating or sustaining role in the continuation of symptoms of ADHD from childhood to adolescence. Stimulant use does not seem to be protective herein.

The two persisting combined type trajectories of ADHD were continuously exposed to higher levels of stress compared to the two less severe remitting ADHD trajectories (i.e., moderate combined type and inattentive type). This can be explained by both heightened sensitivity to and reduced regulation of stress, as these two vulnerabilities hold true particularly for adolescents with combined type ADHD. Children with ADHD enter adolescence with—on average—a less matured prefrontal cortex [27]. Adolescence, in particular, is a period of important changes in brain connectivity within the prefrontal cortex and with other brain regions, supporting the maturation of cognitive control, such as emotion regulation abilities and impulse control abilities [28,29,30]. During adolescence, the developing prefrontal cortex is especially vulnerable to the effects of stress [31]. Our findings support the idea that prefrontal cortex immaturity at the start of adolescence and slower prefrontal maturation during adolescence makes children with ADHD particularly vulnerable in terms of both self-generation of stress and the potentially harmful effects of stress on prefrontal functions of the brain, setting the stage for a persistent and comorbid form of ADHD. Poor socio-economic circumstances and lower IQ may play an additional role herein. 

Of strong interest is the finding that high-stress exposure was associated with two persistent ADHD trajectories with diverging comorbid trajectories: one characterized by persistently elevated irritability, as well as elevated and increasing internalizing problems but without the more extreme reactivity and frustration; the other characterized by severe ongoing emotional regulation problems (irritability, extreme reactivity, frustration) but without the internalizing problems. This finding supports earlier work showing etiological overlap (in cognitive control) between internalizing and externalizing symptoms [32,33]. At the same time, our findings indicate that a subset of children with the most severe persistent ADHD and exposed to high stress are not vulnerable for internalizing problems. Trait impulsivity in young childhood is thought to be one of the driving liabilities to combined-type ADHD among children and is a core predisposing vulnerability to all (adult) externalizing psychopathology [34]. The diverging trajectories of the two most severe ADHD groups in our sample may be explained by the differential presence of early trait anxiety, which is thought to attenuate impulsivity to some extent while representing a vulnerability for progression into more severe internalizing psychiatric outcomes [34]. Indeed, our findings show differences in anxiety/depression, impulsivity, extreme reactivity, and frustration that were apparent at age 11, suggesting that the divergence of both pathways likely started before the onset of adolescence. Trait impulsivity and anxiety are thought to fully manifest as psychiatric symptoms upon interaction with the environment [34]. Chronic stress exposure may be one such potent transactional factor along the ADHD course from young childhood into adulthood. 

Intriguingly, we did not identify subgroups for whom high-stress levels lead to trajectories of internalizing problems or severe emotion dysregulation problems in the absence of ADHD. Only a “mild internalizing” subgroup was found with enhanced but much lower stress levels. This finding may be taken to indicate that stress-exposure and ADHD are even more intertwined than currently acknowledged in the literature which is focused on the role of stress in depression and anxiety. Importantly, the finding should not be interpreted to show the irrelevance of the classic stress–stress cycle. Rather, since we oversampled male participants, this cycle is more likely in our non-sampled screen-negative female participants. It is likely that our identified “mild internalizing” subgroup will develop more severe internalizing problems when they reach their early twenties when depression and several anxiety disorders have their peak incidence. Our findings are suggestive of a different underlying etiology for anxiety-depression with and without ADHD, respectively. We tentatively propose a neurodevelopmental subtype of “internalizing ADHD,” which is different from the adolescent onset of internalizing problems without ADHD. This distinction between “neurodevelopmental” and “traditional” internalizing problems is strongly consistent with a series of recent papers on the role of childhood irritability in depression [35,36]. These authors similarly propose the existence of “two depressions.” Here we confirm the neurodevelopmental route to depression and show that (similar to “typical” i.e., adolescent and young adulthood-onset depression) this route is strongly intertwined with stress exposure.

Our study has strong assets. We applied multivariate latent class growth modeling that captures this heterogeneity in both stress exposure and behavioral symptoms. This approach makes full use of the repeated simultaneous measurement of all variables by parsing subgroups of adolescents with specific forms of continuity and change [15]. In addition, our same age cohort study design is critical for studying the heterogeneity of the ADHD course. Often, ADHD participants of varying ages are combined in a sample, confounding the heterogeneity that exists among same-age individuals with heterogeneity caused by differential development over time. That is, by combining various ages, it is, for example, ignored that hyperactivity in childhood, typical for ADHD at that age, has a different meaning in adolescence, when it is less commonly reported [37]. Our study design of same-age individuals followed over time is very strong in disentangling momentaneous heterogeneity from developmental heterogeneity. 

Our study has limitations as well. The current study of trajectories does not allow inferences of causality; rather our analyses show the strong intertwinement of high-stress exposure with two qualitatively different trajectories of persistent ADHD, both different from remitting ADHD. We regard our study, therefore, as the first necessary step in showing the relevance of stress exposure for the course of ADHD, which has been ignored so far in the literature. Future observational and treatment studies may extend our work by focusing on the causal relations among stress exposure and the symptoms of ADHD over time. As the early stages of a progressive course of ADHD were already apparent in pre-adolescents, we recommend to not only study the potential causal role of stress during adolescence but also pre-adolescence and even pre-onset to ADHD. We propose to study the consequences of stress exposure on ADHD symptoms both at the microlevel, capturing day-to-day causal relations, and at the macrolevel for causal changes over longer periods of time.

Our findings suggest that taking into account stressful environmental factors should be an inherent part of the diagnosis, prognosis, and treatment of ADHD. The clinical profile and level of stress exposure at the start of adolescence are strong markers for what comes next: preadolescents living in chronic stressful conditions who have combined type ADHD symptoms and enhanced anxiety and depression are likely to have persistent ADHD and future exacerbation of anxiety and depression. Conversely, under similar stressful conditions, high parent-rated compared to self-rated emotion regulation problems mark the highly severe and persistent course of both ADHD and emotion dysregulation symptoms. High-quality normative data for instruments that adequately tap these behaviors, derived from pre-adolescents with ADHD may aid clinical thinking about the prognosis. Importantly, the extent to which individuals expose themselves to stressful conditions and how they manage stress are potentially modifiable, thereby providing a therapeutic angle for prevention or interruption of the negative cycle of persistent ADHD and (development of) comorbid conditions.

In sum, we conclude that the stress–psychopathology–stress cycle likely plays an important facilitating and sustaining role in the persistence of ADHD and (development of) comorbid problems from childhood to adolescence. We showed that ADHD combined type with high-stress exposure can either take the form of co-occurring severe and persistent emotion dysregulation (irritability, frustration, extreme reactivity) or persistently high irritability and increased anxiety and depression symptoms. The former trajectory fits well with recent literature that emphasizes that emotion dysregulation is present in a substantial subset of children with ADHD and we show that the course of this group into adulthood is unfavorable. The latter trajectory fits well with a recent emphasis on a neurodevelopmental route to depression. Conversely, low and declining stress exposure and remitting or inattentive ADHD may accumulate towards desistence from both internalizing and externalizing problems. Clinicians may, therefore, include the presence of stressful conditions in the diagnostic process for prognosis and potential prevention or interruption of an adverse ADHD course. 

## 5. Conclusions

- Cross-sectional studies have indicated that individuals with ADHD are on average exposed to more stressful conditions than typically developing individuals.
- Current findings show that stress exposure is strongly intertwined with a persistent course of ADHD between childhood and young adulthood.
- High exposure to stress that peaks in mid-adolescence go jointly with two different persistent courses: combined type ADHD with strong irritability, extreme reactivity, and high frustration and combined type ADHD with elevated and increasing irritability, anxiety, and depression.
- Consideration of stressful conditions should be part of the diagnosis and treatment of ADHD for prognosis as well as potential prevention or interruption of adverse trajectories.

## Figures and Tables

**Table 1:** Characteristics of the seven subgroups^1^.

		Non-ADHD Subgroups			ADHD Subgroups				
Characteristic	Overall	No Problems	Mild Inattention	Mild Internalizing	Moderate Combined	Inattentive	Internalizing Combined	Severe Combined	Wald (df), *p*-value
		N (%)	N (%)	N (%)	N (%)	N (%)	N (%)	N (%)	
**Overall**	1831	389	367	235	267	227	153	193	587 (6), *p* < 0.0001
**Cohort**									174053 (6), *p* < 0.0001
Population	1587	389 (100%)	358 (98%)	235 (100%)	204 (76%)	207 (91%)	115 (75%)	80 (41%)	
Clinical	244	0 (0%)	9 (2%)	0 (0%)	63 (24%)	20 (9%)	38 (25%)	113 (59%)	
**Gender**									480 (6), *p* < 0.0001
Female	583	108 (28%)	68 (19%)	135 (57%)	67 (25%)	65 (29%)	80 (52%)	60 (31%)	
Male	1248	281 (72%)	299 (81%)	100 (43%)	200 (75%)	162 (71%)	73 (48%)	133 (69%)	
**SES**									220 (6), *p* < 0.0001
<25%	474	76 (20%)	88 (24%)	56 (24%)	80 (30%)	55 (24%)	56 (38%)	63 (33%)	
25-75%	890	167 (44%)	186 (51%)	110 (48%)	134 (51%)	117 (52%)	72 (48%)	104 (55%)	
>75%	437	138 (36%)	89 (25%)	64 (28%)	50 (19%)	53 (24%)	21 (14%)	22 (12%)	
**Ethnicity**									31 (6), *p* < 0.0001
Western	1659	351 (90%)	333 (91%)	207 (88%)	247 (93%)	207 (91%)	132 (87%)	182 (95%)	
Non-western^2^	170	38 (10%)	34 (9%)	29 (12%)	20 (7%)	20 (9%)	20 (13%)	9 (5%)	
**IQ**									206 (6), *p* < 0.0001
Low	576	92 (24%)	108 (30%)	53 (23%)	116 (44%)	69 (30%)	62 (41%)	76 (40%)	
Middle	675	145 (37%)	135 (37%)	94 (40%)	97 (36%)	87 (38%)	48 (31%)	69 (36%)	
High	574	152 (39%)	121 (33%)	88 (37%)	53 (20%)	71 (31%)	43 (28%)	46 (24%)	
**ADHD**									1096 (6), *p* < 0.0001
Negative screen	1222	382 (98%)	315 (86%)	224 (95%)	86 (32%)	147 (65%)	57 (37%)	11 (6%)	
Positive screen	365	7 (2%)	43 (12%)	10 (4%)	118 (44%)	60 (26%)	58 (38%)	69 (36%)	
Clinical^3^	244	0 (0%)	9 (2%)	1 (1%)	63 (24%)	20 (9%)	38 (25%)	113 (59%)	

Note. Frequencies based on posterior mean probabilities rounded to the nearest whole number. ^1^ All characteristics differed statistically significant across subgroups at p < 0.0001 and are discussed in the supplementary text in more detail. ^2^ At least one parent born in a non-western country. ^3^ Adolescents with a clinical diagnosis of ADHD (n = 244) are the same participants as those listed under the clinical cohort.

## Supplementary Materials

The following are available online at https://www.mdpi.com/2077-0383/8/11/1824/s1.

## References

[1] Hartman C.A., Geurts H.M., Franke B., Buitelaar J.K., Rommelse N.N. (2016). Changing ASD-ADHD symptom co-occurrence across the lifespan with adolescence as crucial time window: Illustrating the need to go beyond childhood. Neurosci. Biobehav. Rev..

[2] Franke B., Michelini G., Asherson P., Banaschewski T., Bilbow A., Buitelaar J.K., Cormand B., Faraone S.V., Ginsberg Y., Haavik J. (2018). Live fast, die young? A review on the developmental trajectories of ADHD across the lifespan. Eur. Neuropsychopharmacol..

[3] Grant K.E., Compas B.E., Thurm A.E., McMahon S.D., Gipson P.Y. (2004). Stressors and child and adolescent psychopathology: Measurement issues and prospective effects. J. Clin. Child Adolesc. Psychol..

[4] Biederman J., Milberger S., Faraone S.V., Kiely K., Guite J., Mick E., Warburton R., Reed E., Davis S.G. (1995). Impact of adversity on functioning and comorbidity in children with attention-deficit hyperactivity disorder. J. Am. Acad. Child Adolesc. Psychiatry.

[5] Björkenstam E., Björkenstam C., Jablonska B., Kosidou K. (2018). Cumulative exposure to childhood adversity, and treated attention deficit/hyperactivity disorder: A cohort study of 543 650 adolescents and young adults in Sweden. Psychol. Med..

[6] Blazer D., Hughes D., George L.K. (1987). Stressful life events and the onset of a generalized anxiety syndrome. Am. J. Psychiatry.

[7] Campo J.V. (2012). Annual Research Review: Functional somatic symptoms and associated anxiety and depression–developmental psychopathology in pediatric practice. J. Child Psychol. Psychiatry.

[8] Kendler K.S., Karkowski L.M., Prescott C.A. (1998). Stressful life events and major depression: Risk period, long-term contextual threat, and diagnostic specificity. J. Nerv. Ment. Dis..

[9] Larsson H., Dilshad R., Lichtenstein P., Barker E.D. (2011). Developmental trajectories of DSM-IV symptoms of attention-deficit/hyperactivity disorder: Genetic effects, family risk and associated psychopathology. J. Child Psychol. Psychiatry.

[10] Barkley R.A. (2015). Emotional dysregulation is a core component of ADHD. Attention-Deficit Hyperactivity Disorder: A Handbook for Diagnosis and Treatment.

[11] Bunford N., Evans S.W., Wymbs F. (2015). ADHD and emotion dysregulation among children and adolescents. Clin. Child Fam. Psychol. Rev..

[12] Wheeler Maedgen J., Carlson C.L. (2000). Social functioning and emotional regulation in the attention deficit hyperactivity disorder subtypes. J. Clin. Child Psychol..

[13] Melnick S.M., Hinshaw S.P. (2000). Emotion regulation and parenting in AD/HD and comparison boys: Linkages with social behaviors and peer preference. J. Abnorm. Child Psychol..

[14] Oldehinkel A.J., Rosmalen J.G., Buitelaar J.K., Hoek H.W., Ormel J., Raven D., Reijneveld S.A., Veenstra R., Verhulst F.C., Vollebergh W.A. (2015). Cohort Profile Update: The TRacking Adolescents’ Individual Lives Survey (TRAILS). Int. J. Epidemiol..

[15] Muthén B., Kaplan D. (2004). Latent variable analysis: Growth mixture modeling and related techniques for longitudinal data. Handbook of Quantitative Methodology for the Social Sciences.

[16] Costello A., Edelbrock C., Kalas R., Kessler M., Klaric S.A. (1982). Diagnostic Interview Schedule for Children (DISC).

[17] Steenhuis M.P., Serra M., Minderaa R.B., Hartman C.A. (2009). An internet version of the Diagnostic Interview Schedule for Children (DISC-IV): Correspondence with paper and pencil version using the ADHD section. Psychol. Assess..

[18] Achenbach T.M., Ruffle T.M. (2000). The Child Behavior Checklist and related forms for assessing behavioral/emotional problems and competencies. Pediatr. Rev..

[19] Hartman C.A., Luteijn E., Serra M., Minderaa R. (2006). Refinement of the Children’s Social Behavior Questionnaire (CSBQ): An instrument that describes the diverse problems seen in milder forms of PDD. J. Autism Dev. Disord..

[20] Putnam S.P., Ellis L.K., Rothbart M.K., Eliasz A., Angleitner A. (2001). The structure of temperament from infancy through adolescence. Advances/Proceedings in Research on Temperament.

[21] Sattler J.M. (1992). Assessment of Children.

[22] Nagin D.S., Tremblay R.E. (2001). Analyzing developmental trajectories of distinct but related behaviors: A group-based method. Psychol. Methods.

[23] Lanza S.T., Collins L.M., Lemmon D., Schafer J.L. (2007). PROC LCA: A SAS procedure for latent class analysis. Struct. Equ. Model..

[24] Yang C. (2006). Evaluating latent class analysis models in qualitative phenotype identification. Comput. Stat. Data Anal..

[25] Vermunt J.K. (2010). Latent class modeling with covariates: Two improved three-step approaches. Political Anal..

[26] Vermunt J.K., Magidson J. (2016). Technical Guide for Latent GOLD 5.1: Basic, Advanced and Syntax.

[27] Cubillo A., Halari R., Smith A., Taylor E., Rubia K. (2012). A review of fronto-striatal and fronto-cortical brain abnormalities in children and adults with Attention Deficit Hyperactivity Disorder (ADHD) and new evidence for dysfunction in adults with ADHD during motivation and attention. Cortex.

[28] Burnett Heyes S., Jih Y.R., Block P., Hiu C.F., Holmes E.A., Lau J.Y. (2015). Relationship reciprocation modulates resource allocation in adolescent social networks: Developmental effects. Child Dev..

[29] Koolschijn P.C.M., Crone E.A. (2013). Sex differences and structural brain maturation from childhood to early adulthood. Dev. Cogn. Neurosci..

[30] Ladouceur C.D. (2012). Neural systems supporting cognitive-affective interactions in adolescence: The role of puberty and implications for affective disorders. Front. Integr. Neurosci..

[31] Lupien S.J., McEwen B.S., Gunnar M.R., Heim C. (2009). Effects of stress throughout the lifespan on the brain, behaviour and cognition. Nat. Rev. Neurosci..

[32] Caspi A., Houts R.M., Belsky D.W., Goldman-Mellor S.J., Harrington H., Israel S., Moffitt T.E. (2014). The p factor: One general psychopathology factor in the structure of psychiatric disorders?. Clin. Psychol. Sci..

[33] Bloemen A.J.P., Oldehinkel A.J., Laceulle O.M., Ormel J., Rommelse N.J., Hartman C.A. (2018). The association between executive functioning and psychopathology: General or specific?. Psychol. Med..

[34] Beauchaine T.P., Zisner A.R., Sauder C.L. (2017). Trait impulsivity and the externalizing spectrum. Ann. Rev. Clin. Psychol..

[35] Addicoat A., Thapar A.K., Riglin L., Thapar A., Collishaw S. (2019). Adult mood problems in children with neurodevelopmental problems: Evidence from a prospective birth cohort followed to age 50. Soc. Psychiatry Psychiatr. Epidemiol..

[36] Eyre O., Hughes R.A., Thapar A.K., Leibenluft E., Stringaris A., Davey Smith G., Stergiakouli ECollishaw S., Thapar A. (2019). Childhood neurodevelopmental difficulties and risk of adolescent depression: The role of irritability. J. Child Psychol. Psychiatry.

[37] McGough J.J., Barkley R.A. (2004). Diagnostic controversies in adult attention deficit hyperactivity disorder. Am. J. Psychiatry.

