# Supplementary material for: Stress Exposure and the Course of ADHD from Childhood to Young Adulthood: Comorbid Severe Emotion Dysregulation or Mood and Anxiety Problems
Source: J Clin Med. 2019 Nov 1;8(11):1824. doi: 10.3390/jcm8111824 (PMC6912831; doi:10.3390/jcm8111824)
Supplement: Supplementary file 1 [file jcm-08-01824-s001.pdf]

**T1 11.1 years**	**T2 13.6 years**	**T3 16.3 years**	**T4 19.1 years**
IQ			
attention problems	attention problems	attention problems	
hyperactivity/impulsivity	hyperactivity/impulsivity	hyperactivity/impulsivity	
anxiety	anxiety	anxiety	anxiety
depression	depression	depression	depression
unexplained somatic complaints	unexplained somatic complaints	unexplained somatic complaints	unexplained somatic complaints
irritability	irritability	irritability	irritability
extreme reactivity	extreme reactivity	extreme reactivity	extreme reactivity
frustration		frustration	frustration
stress exposure	stress exposure	stress exposure	stress exposure

Supplemental Figure 1. Overview of the measurements per measurement wave.

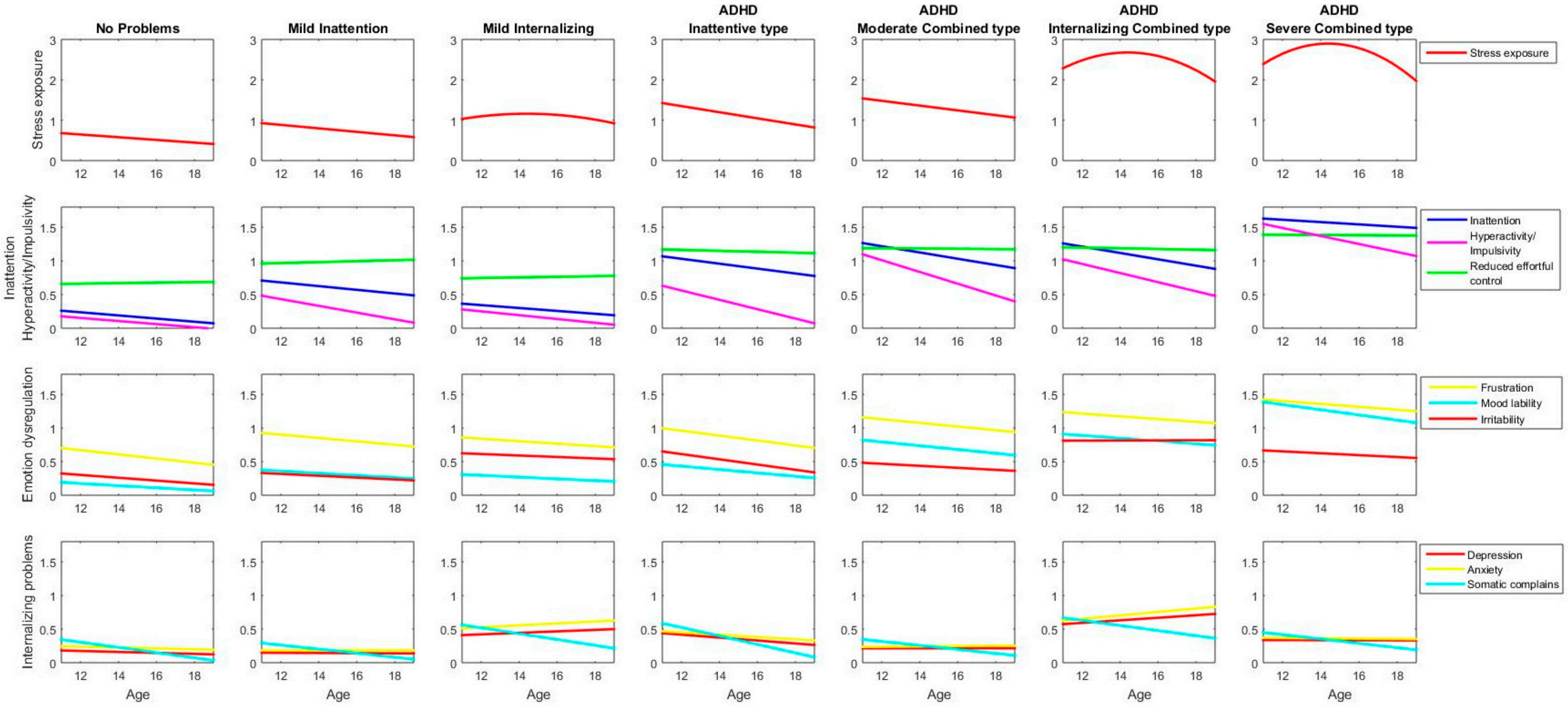

Supplemental Figure 2 (unstandardized results). Results of the multivariate latent class growth analysis identifying seven subgroups that differed in the course of stress exposure, core ADHD symptoms, effortful control and internalizing and emotion regulation problems. Unstandardized estimates are plotted for each subgroup (left to right) on trajectories across the four domains (top to bottom).

Journal of
Clinical Medicine

MDPI

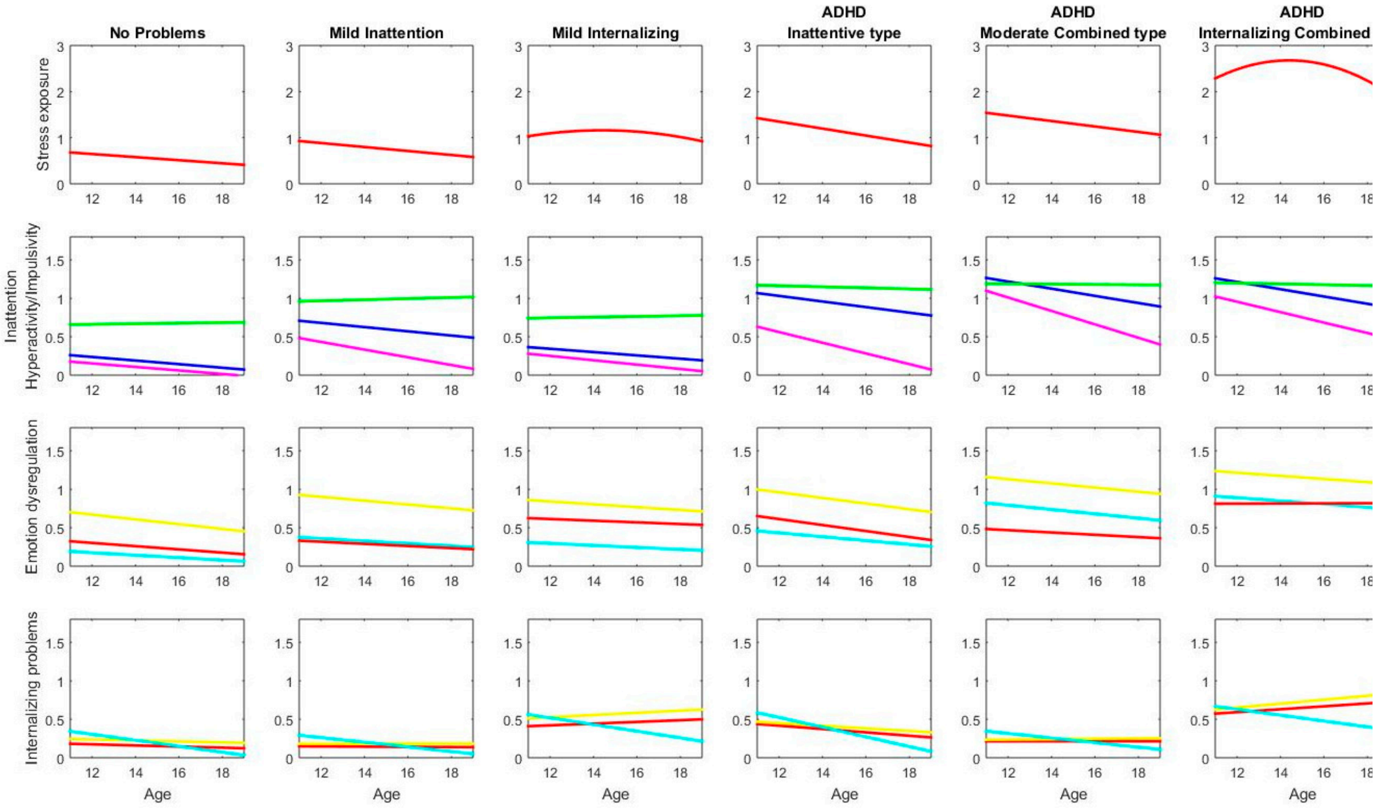

Supplemental Figure 2 (unstandardized results). Results of the multivariate latent class growth analysis identifying seven subgroups that differed in the course of stress exposure, core ADHD symptoms, effortful control and internalizing and emotion regulation problems. Unstandardized estimates are plotted for each subgroup (left to right) on trajectories across the four domains (top to bottom).
